# Inflammatory niches as spatial drivers of disease mechanisms and targets for personalized treatment

**DOI:** 10.1111/jdv.70170

**Published:** 2025-11-07

**Authors:** Rundong Jiang, Zhiqin Fang, Lam C. Tsoi, Johann E. Gudjonsson

**Affiliations:** ^1^ Department of Dermatology University of Michigan Medical School Ann Arbor Michigan USA; ^2^ Graduate Program in Immunology University of Michigan Ann Arbor Michigan USA; ^3^ Department of Dermatology, Division of Life Sciences and Medicine, The First Affiliated Hospital of USTC University of Science and Technology of China Hefei Anhui China

**Keywords:** inflammatory skin diseases, single‐cell RNA sequencing, spatial transcriptomics

## Abstract

Disease states are increasingly recognized as being shaped by spatially organized inflammatory niches, in which immune and non‐immune cells coordinate diverse biological processes, including tissue development, homeostasis and pathology. The activation and composition of specific inflammatory responses, as well as the signalling pathways involved, are highly context‐ and location‐dependent. Recent technological advances—particularly the integration of single‐cell sequencing with spatial profiling—have greatly enhanced our ability to dissect these processes at a molecular level. Such approaches have revealed disease‐specific mechanisms, including IL‐36‐driven feed‐forward amplification in the upper layers of psoriatic epidermis, the complex inflammatory architecture of hidradenitis suppurativa and T‐cell–basal keratinocyte interactions in lichen planus. Continued investigation of inflammatory niches across diseases will deepen our understanding of their pathogenic mechanisms, enable the identification of patient‐specific cellular networks and pave the way for more personalized therapeutic strategies.

## Graphical abstract

This study provides a comprehensive review of how spatial transcriptomics reveals disease‐specific inflammatory niches across multiple skin disorders, highlighting key immune–stromal, neuro–immune and metabolic interactions that were previously unappreciated in non‐spatial analyses.

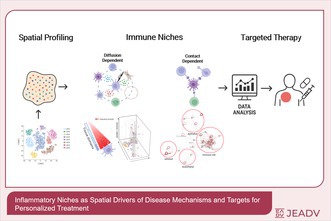

**Why was the study undertaken?**

- To investigate how spatially organized inflammatory niches shape disease pathogenesis by integrating single‐cell and spatial transcriptomic technologies, aiming to uncover location‐ and context‐specific immune mechanisms across inflammatory skin diseases.

**What does this study add?**

- This study provides a comprehensive review of how spatial transcriptomics reveals disease‐specific inflammatory niches across multiple skin disorders—including psoriasis, atopic dermatitis, lichen planus, hidradenitis suppurativa, vitiligo and systemic sclerosis—highlighting key immune–stromal, neuro‐immune and metabolic interactions that were previously unappreciated in non‐spatial analyses.

**What are the implications of this study for disease understanding and/or clinical care?**

- By uncovering spatially organized pathogenic circuits and identifying niche‐specific molecular targets, this work lays the groundwork for patient stratification and spatially guided personalized therapies in chronic inflammatory skin diseases, moving towards precision dermatology.

## INTRODUCTION

Over the past two decades, our understanding of cutaneous immune responses has expanded dramatically, driving the development of targeted therapies that have transformed the management of inflammatory skin diseases such as psoriasis and atopic dermatitis (AD).^1^, ^2^, ^3^ In particular, comprehensive cellular and molecular profiling has uncovered aberrant activation of the IL‐23/IL‐17 axis in psoriasis^4^ and the IL‐4/IL‐13 axis in AD^5^ as key pathogenic drivers, thereby enabling the advent and clinical success of targeted biologic therapies. Notably, these diseases are characterized by intricate spatial tissue architectures and pronounced cellular heterogeneity, both of which are central to their pathogenesis and clinical presentation.

In contrast to systemic diseases in which circulating immune factors predominate, cutaneous inflammation is tightly compartmentalized, structured across the epidermis, dermis and adnexal units, with discrete microenvironments that orchestrate distinct immune responses. In psoriasis, epidermal tissue‐resident memory T cells (T_RM_), particularly CD8^+^ T_RM_ cells, persist long after clinical resolution and are implicated in site‐specific disease recurrence.^6^ Similarly, in AD, the transition from acute to chronic disease is accompanied by dynamic spatial remodelling of immune responses. Notably, memory Th2 cells can persist long‐term even in patients receiving treatment, potentially contributing to disease chronicity and relapse.^7^ In parallel, barrier dysfunction in keratinocytes plays a pivotal role in initiating immune activation and sustaining inflammation, further reinforcing the spatially compartmentalized nature of the disease.^8^

Although multiple inflammatory circuits have been implicated in various inflammatory skin diseases, including TNF, IL‐23/IL‐17, IL‐22, IL‐4/IL‐13, TGF‐β and IFN‐γ pathways, distinguishing disease‐specific molecular abnormalities has historically been challenging using bulk transcriptomic profiling, which inherently obscures cell type‐specific transcriptional dysregulation.^9^ A striking example of this limitation is seen in diseases such as hidradenitis suppurativa (HS) and psoriasis: despite sharing similar inflammatory pathways, particularly TNF and IL‐23/IL‐17 signalling,^10^ bulk data fail to explain why biologic therapies targeting TNF‐α or IL‐17A show markedly lower clinical efficacy in HS compared to psoriasis.^11^, ^12^ This therapeutic discrepancy highlights the urgent need for approaches that can resolve the cellular contexts of inflammation, offering deeper insights into disease‐specific pathogenic mechanisms.

To address this limitation, single‐cell RNA sequencing (scRNA‐seq) has emerged as a powerful technology by enabling the dissection of gene expression programs at single‐cell resolution, even from small tissue samples.^13^ This approach has uncovered previously unrecognized disease factors across multiple skin inflammatory pathologies.^14^, ^15^, ^16^ However, a key limitation of scRNA‐seq lies in its requirement for tissue dissociation, which results in the loss of spatial information, obliterating the three‐dimensional architecture and microniches that are critical for understanding cell–cell interactions and the tissue microenvironment in both health and disease.

**FIGURE 1:**
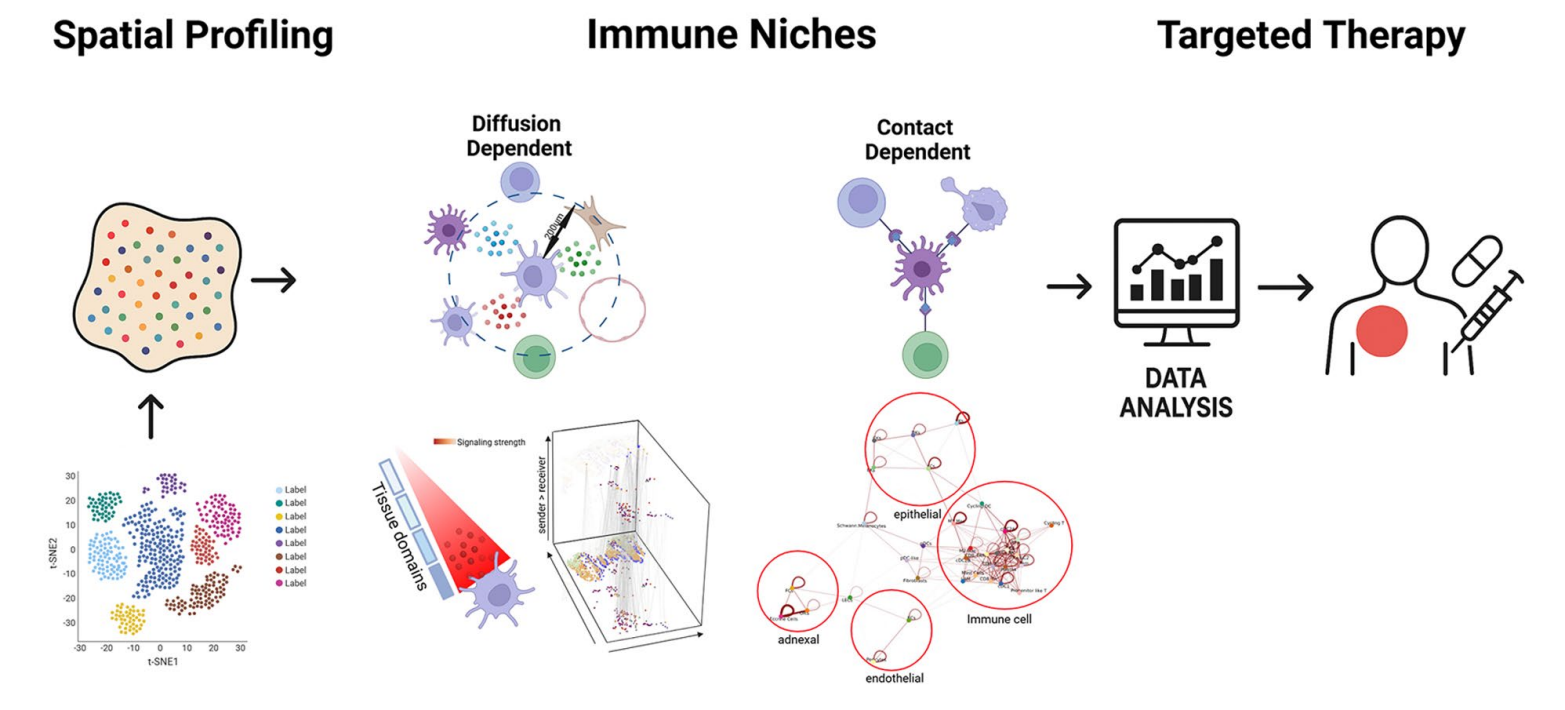
Spatially resolved analysis of skin cellular interactions for precision medicine. Integration of single‐cell and spatial transcriptomic data enables improved cell‐type annotation and identification of disease‐relevant biological mechanisms. This approach reveals immune niches, which may be diffusion‐dependent (e.g. cytokine/chemokine secretion) or contact‐dependent (e.g. receptor–ligand or adhesion interactions). Incorporating these spatially organized interactions into analyses helps define disease‐ and patient‐specific inflammatory patterns, thereby informing personalized therapeutic strategies.

Spatial transcriptomics (ST) was developed to address this limitation by enabling in situ visualization and quantitative analysis of transcriptomes within intact tissue sections.^17^ Although current ST approaches generally cannot yet simultaneously achieve the single‐cell resolution and transcriptomic depth provided by scRNA‐seq, they provide valuable insights into the spatial distribution of gene expression and the localization of distinct cell populations within their native niches.^14^, ^18^ Importantly, ST can highlight regions enriched for specific signalling pathways and identify ligand‐receptor interactions mediating local cellular communication.^19^ When integrated with scRNA‐seq data, spatial transcriptomics enables the mapping of transcriptionally defined single‐cell populations back to their native tissue context, providing a more comprehensive view of cellular organization and interaction networks.^14^, ^20^ Such integrative approaches are poised to deepen our understanding of how spatially organized immune and non‐immune cells contribute to tissue development, homeostasis and inflammatory disease pathogenesis (Figure 1).

## APPLICATION OF ST IN INFLAMMATORY SKIN DISEASES

### Psoriasis

Psoriasis is a common chronic inflammatory skin disorder characterized by a dual immune signature: autoimmune‐associated pathways dominated by IFN‐γ and IL‐17, mixed with autoinflammatory responses driven by elevated IL‐36 expression.^21^ Spatial transcriptomics has provided unprecedented insights into the layered organization of inflammatory circuits within lesional skin. Spatial mapping of IL‐17‐driven keratinocyte programs has revealed that keratinocyte hyperproliferation and aberrant inflammatory responses are not uniformly distributed across the epidermis. Instead, these transcriptional programs are preferentially enriched within the suprabasal layers,^22^ forming discrete regions in close proximity to immune cell niches. These niches are predominantly composed of IL‐17 producing Th17 cells and mostly epidermal CD8^+^ T_RM_ cells,^14^ which persist at the dermal–epidermal junction even after clinical remission and are thought to serve as local reservoirs driving site‐specific disease recurrence. In addition, spatial analyses have uncovered secondary cytokine responses, such as IL‐36 responses, which are primarily observed in the supraspinous keratinocytes located in the outermost epidermal layer,^14^ suggesting a feed‐forward loop in which keratinocytes amplify inflammation by recruiting neutrophils and activating myeloid populations in superficial pustule.^23^ Moreover, integrative analyses combining ST and scRNA‐seq have uncovered previously unrecognized fibroblast subsets characterized by distinct transcriptional signatures. These fibroblasts co‐express high levels of collagen genes (e.g. COL1A1, COL3A1) together with pro‐inflammatory cytokines (e.g. CCL19, IL‐33, IL‐34) and are preferentially localized at the tips of the dermal papillae. This spatially restricted fibroblast population forms fibro‐inflammatory hubs that orchestrate active immune responses, recruiting and positioning multiple immune cell lineages, including T cells, myeloid cells and B cells.^18^ Together, these findings underscore how spatially resolved transcriptomics, especially when combined with single‐cell data, enable the reconstruction of psoriasis‐specific microenvironments and highlight potential therapeutic targets within spatially confined pathogenic niches.

### Atopic dermatitis (AD)

Atopic dermatitis (AD) is a chronic inflammatory skin disorder characterized by impaired epidermal barrier function and aberrant immune activation. Immunologically, AD is predominantly driven by Th2 and Th22 cytokine pathways, which orchestrate the hallmark eczematous inflammation.^24^ ST has provided critical insights into how immune polarization, barrier dysfunction and metabolic alterations converge in specific tissue niches. First, ST mapping has revealed a Th2‐dominant immune landscape characterized by IL‐4/IL‐13‐producing T cells concentrated near areas of barrier disruption,^25^ where keratinocytes exhibit a loss of function of barrier structural proteins such as filaggrin and loricrin.^26^ This spatial juxtaposition highlights that keratinocyte barrier defects are not diffuse but localized to immune hotspots, directly linking structural impairment to Th2‐skewed inflammation. In addition, ST has also uncovered spatial heterogeneity in lipid metabolism pathways, identifying focal regions with diminished ceramide and free fatty acid biosynthesis that colocalize with sites of intense Th2 cytokine signalling,^27^, ^28^ suggesting a metabolic–immune axis underlying chronic barrier dysfunction. Third, integrative ST and scRNA‐seq analyses have revealed that COL18A1‐expressing fibroblasts at the dermal–epidermal interface of AD skin show marked upregulation of extracellular matrix genes (*COL6A5*, *COL4A1*, *TNC*) and the chemokine *CCL19*. These fibroblasts form fibro‐immune hubs that spatially align with infiltrating Th2 cells, *CCL18*‐producing M2 macrophages and *LAMP3*‐expressing dendritic cells, thereby sustaining chronic inflammation. Collectively, these spatially resolved insights demonstrate that AD pathology arises from localized crosstalk among keratinocytes, immune cells and fibroblasts, coupled with regional metabolic dysregulation that was previously masked in bulk or dissociated single‐cell analyses.

### Lichen planus

Lichen planus (LP) is a chronic inflammatory condition typified by purple, polygonal, pruritic papules and plaques.^29^ LP is a T‐cell‐mediated disease with IFN‐γ established as a key mediator in pathogenesis.^30^ Although only limited spatial transcriptomics data have been published to date, prior clinical studies examining baricitinib treatment have reported robust infiltration of myeloid cells and CXCL13^+^ CD8^+^ T cells in the upper dermis, which are closely associated with basal keratinocyte injury.^31^ Consistently, our unpublished spatial analyses of both cutaneous and mucosal LP provide further insights into the hallmark interface dermatitis, revealing that CD8^+^ cytotoxic T cells are spatially concentrated along this interface, colocalizing with apoptotic keratinocytes and expressing high levels of cytolytic mediators such as *GZMB*, *PRF1* and *IFNG*. Furthermore, our integrative analyses have uncovered previously underappreciated myeloid‐T‐cell crosstalk within these immune foci: classical type 2 dendritic cells subset A (cDC2A) are positioned adjacent to CXCL13^+^ CD8^+^ T cells, expressing IL‐15 and costimulatory molecules that amplify CD8^+^ T‐cell recruitment and activation. Intriguingly, another study integrating ST with scRNA‐seq revealed that LP lesions harbour not only a dominant type 1 immune response but also significant type 2 inflammatory activity,^32^ thereby providing a mechanistic rationale for the observed clinical benefit of IL‐4Rα blockade in this disease.

### Hidradenitis suppurativa (HS)

Hidradenitis suppurativa (HS) is a chronic inflammatory skin disease characterized by recurrent painful nodules and abscesses arising from hair follicles, most commonly affecting the axillae and groin.^33^ Immunopathologically, HS lesions exhibit heightened expression of pro‐inflammatory cytokines released from T cells and myeloid cells, including IL‐1β, IL‐17 and TNF‐α.^34^ ST analyses have revealed underappreciated inflammatory remodelling within HS sinus tracts: beyond T cells and myeloid cells, lesions contain B‐cell aggregates and ectopic germinal center‐like structures enriched for B‐cell activation and class‐switch recombination signatures,^35^ indicating ongoing local antibody responses within these chronic inflammatory niches. Additionally, ST mapping has revealed pronounced tertiary lymphoid structures (TLSs) not only within chronic HS lesions but also in early‐stage disease.^36^ Within these TLSs, CXCL13^+^ CD4^+^ peripheral helper T (Tph) cells are markedly expanded and exhibit robust expression of genes involved in TLS organization and B‐cell maturation.^35^ Moreover, integrative ST analysis has identified fibro‐inflammatory niches at the periphery of sinus tracts in HS lesions. Notably, two fibroblast subtypes, SFRP4^+^ and CXCL13^+^ fibroblasts, play pivotal roles in orchestrating this compartmentalized immune response with dendritic cells. More importantly, these pathogenic fibroblasts exhibit activation of the Hippo signalling pathway, which drives extensive fibrosis in HS,^15^ highlighting a potential therapeutic avenue. Together, these spatially resolved insights provide a framework for understanding how localized immune–stromal interactions shape HS pathology and may inform more precise therapeutic targeting of pathogenic niches.

### Vitiligo

Vitiligo is a common autoimmune skin disease, characterized by white patches on skin and attributed to melanocytes attacked by autoreactive CD8^+^ T cells.^37^ Recent advances in spatial transcriptomics, complemented by single‐cell analyses, have delineated the complex immune–epithelial–neuronal landscape of vitiligo. Impressively, a previous study demonstrated that autoreactive CD8^+^ tissue‐resident memory T (T_RM_) cells accumulate at the dermal–epidermal junction in close apposition to melanocytes and stressed keratinocytes, expressing cytotoxic mediators (*IFNG*, *GZMB*, *PRF1*) that orchestrate melanocyte destruction.^6^ Concomitantly, keratinocytes exhibit barrier dysfunction and pro‐inflammatory signalling, reinforcing T‐cell activation.^38^ Furthermore, neuro‐immune crosstalk emerges as a pivotal pathogenic axis: nociceptor‐derived calcitonin gene‐related peptide (CGRP) engages CALCRL^+^ cDC1 cells, boosting antigen presentation and priming of cytotoxic CD8^+^ T cells. Pharmacologic blockade of this CGRP–cDC1 pathway ameliorates depigmentation in both murine models and preliminary human studies.^39^ This neuron‐immune crosstalk was further highlighted by ST profiling of halo nevi, a condition pathophysiologically linked to vitiligo. The study uncovered aberrant melanocyte‐derived metabolites, particularly uridine diphosphate glucose (UDP‐G), which co‐localize with dendritic cell infiltrates and trigger chemokine production such as *CXCL9/10* and *IL‐23*.^40^ This metabolic–immune interface overlaps with regions of oxidative stress and regulated cell death pathways, such as pyroptosis and ferroptosis,^41^ providing a novel layer of insight into vitiligo pathogenesis.

### Systemic sclerosis

Systemic sclerosis (SSc) is a debilitating autoimmune‐driven connective tissue disease defined by multisystem fibrosis, persistent autoimmunity, and prominent microvasculopathy affecting the skin and visceral organs.^42^ These pathological processes exhibit marked spatial heterogeneity, increasingly elucidated by integrative ST analyses. First, integrated ST and single‐cell analyses delineate fibroblast heterogeneity beyond classical paradigms: subpopulations such as SFRP2^+^ reticular dermis fibroblasts, CCL19^+^ non‐perivascular fibroblasts and COL8A1^+^ fibroblasts occupy distinct dermal niches and exhibit divergent profibrotic or proinflammatory transcriptional programs.^43^ Notably, COL8A1^+^ fibroblasts emerge as key effectors of extracellular matrix (ECM) deposition and display intense interactions with macrophages and B cells, correlating with disease progression and skin thickening.^20^, ^43^ Moreover, ST analyses have also identified fibroblast‐macrophage crosstalk as a hallmark feature of SSc lesions. Macrophage‐rich fibrotic niches co‐localize with distinct fibroblast subsets, orchestrating TGF‐β and JAK–STAT signalling hubs that strongly correlate with clinical severity and modified Rodnan skin scores. Notably, the ACKR3 (CXCL12 decoy receptor)–CXCL12/CXCR4 axis, highly enriched in myofibroblast progenitors, regulates proinflammatory macrophage recruitment, and pharmacologic blockade of this pathway attenuates fibrosis in preclinical models, highlighting its potential as a therapeutic target.^44^ Finally, multi‐omic integration highlights the Hippo‐YAP/TAZ pathway as a central driver of both myofibroblast differentiation and endothelial‐to‐mesenchymal transitioning (EndoMT) programs. Hippo effectors (*CTGF, CYR61*) rise along pseudotime trajectories from progenitor fibroblasts to COL8A1^+^ myofibroblasts, and pathway inhibition mitigates fibrosis in experimental models, nominating it as a therapeutic target.^20^ Together, these spatial insights redefine SSc as a disorder of compartmentalized fibro‐immune‐vascular interactions, providing a framework for biomarker‐guided stratification and targeted antifibrotic strategies.

## EXPLORING HOST–MICROBIOME AND METABOLISM THROUGH ST

### Spatial microbial–immune interactions

The skin is a complex and dynamic ecosystem colonized by a diverse array of microorganisms, including bacteria, archaea, fungi and viruses.^45^ While these commensal communities are essential for maintaining skin homeostasis, abnormal host–microbiome interactions can contribute to the pathogenesis of various inflammatory skin diseases, such as atopic dermatitis and hidradenitis suppurativa.^5^, ^46^ Although ST has revolutionized our understanding of immune cell architecture and inflammatory circuits in tissue, its application to the study of host–microbial interactions remains nascent, particularly in skin diseases. These observations highlight an urgent need to spatially resolve host–microbe interactions within lesional skin to uncover their functional implications.

Recent work in tumour tissues provides a compelling proof of concept for spatially mapping microbial content within human tissue architecture. Through applying the ST platform (10× Visium) to colorectal cancer and oral squamous cell carcinoma samples, researchers successfully detected and resolved microbial RNA within complex tissue contexts.^47^ These bacterial communities colonize highly localized microniches characterized by poor vascularization, immunosuppressive features and preferential association with malignant cells exhibiting reduced proliferative activity compared to bacteria‐negative tumour regions. Notably, spatial resolution enabled the identification of cell‐associated bacteria and their interacting host cell types, revealing profound transcriptional reprogramming in these regions, including enrichment of pathways related to inflammation, metastasis, DNA damage repair and cellular dormancy. These findings demonstrate the feasibility and power of spatial approaches to interrogate host–microbiome interactions in situ, laying essential groundwork for future studies in non‐cancerous inflammatory diseases, where microbial dysbiosis has been implicated but remains poorly mapped in spatial terms.

### Spatial metabolic programs

Spatial approaches already begun to illuminate the metabolic reprogramming that occurs in inflammatory skin diseases. Recent studies have shown that metabolic programs are not uniformly distributed but rather display spatial restriction corresponding to distinct cellular neighbourhoods.

Examples include recent ST studies, which have uncovered distinct yet overlapping metabolic landscapes in AD and psoriasis, particularly within sebaceous glands and immune‐enriched niches. In both diseases, sebaceous glands exhibit expression of lipid metabolism and transport genes, including *ALOX15B*, *APOC1*, *FABP7*, *FADS1/2*, *FASN*, *PPARG* and *RARRES1*,^18^ suggesting a core role in skin homeostasis. However, AD lesions preferentially express Th2‐associated lipid modulatory genes such as *HSD3B1*, along with *ACAD8*, *FADS6* and *EBP*, aligning with its Th2‐dominant inflammation. Conversely, psoriasis lesions display stronger signatures of inflammation‐related spatially variable genes like *SERPINF1* and *DDX58* as well as psoriasis‐specific pathways involving lipid metabolism, neutrophil degranulation and antimicrobial peptides.^28^ In addition, other metabolic pathways, including sphingolipid metabolism, oxidative phosphorylation and glycolysis, were also enhanced in psoriasis‐specific epithelia.^18^ These findings emphasize that spatially restricted metabolic programs not only reflect disease‐specific immune circuits but also highlight potential metabolic‐immune checkpoints contributing to chronic skin inflammation.

Furthermore, ST mapping of hypoxia‐inducible factor 1 (*HIF1A*) alongside oxidative stress‐associated genes such as *HMOX1*, *SOD2* and *NOS2* has delineated hypoxic and ROS‐enriched niches within inflamed skin. These metabolic microenvironments are spatially aligned with γδ T17 cell dominated regions in psoriasis,^48^ and cytotoxic T‐cell infiltration zones in cutaneous lupus erythematosus.^49^ Notably, such metabolic–immune interfaces have been further linked to pyroptosis and ferroptosis,^50^ suggesting that localized energy imbalance not only drives immune activation but also contributes to epithelial barrier dysfunction.

## FUTURE DIRECTIONS AND OMIC INTEGRATION

Despite the rapidly growing interest in ST and its unique ability to preserve spatial context, several limitations remain. Notably, the detection sensitivity of current ST platforms still lags behind that of scRNA‐seq. The average captured genes for most spatial platforms are less than 500 in contrast to 2500–3500 per cell in single‐cell sequencing. Furthermore, unlike scRNA‐seq, many spatial profiling technologies do not capture the full transcriptomic landscape at single‐cell resolution, often restricting analyses to a predefined panel of genes or capturing only partial transcriptomes.^51^ Therefore, although ST offers powerful insights into the spatial organization of gene expression, its current limitations necessitate integration with complementary omics approaches, such as scRNA‐seq, proteomics and epigenomic profiling, to achieve more robust and comprehensive biological interpretations. For example, coupling ST with scRNA‐seq facilitates cell‐type annotation and spatial localization, enabling precise mapping of transcriptional programs to their anatomical niches.^52^ Moreover, the incorporation of spatial proteomics and metabolomics adds critical functional layers to spatial transcriptomics, enabling the delineation of how localized metabolic reprogramming influences immune cell recruitment, activation and tissue remodelling.^53^ Furthermore, integrating chromatin accessibility data, such as spatial ATAC‐seq and spatial‐CUT&Tag,^54^, ^55^ provides critical insights into the epigenetic and transcriptional landscapes that regulate cellular differentiation dynamics within skin tissue. These epigenetic approaches will further enhance our understanding of spatial gene regulation under both physiological and pathological conditions. Collectively, these multimodal approaches hold the potential to uncover spatially confined checkpoints that may serve as novel therapeutic targets for inflammatory skin diseases in the near future.

In parallel, advances in artificial intelligence (AI) and computational modelling are enabling the reconstruction of 3D tissue architecture and cellular communication networks from spatial omics data, identifying key cell types and states linked to diagnostic and prognostic features.^56^ Such computational frameworks are particularly impactful in the field of precision dermatology, where spatial heterogeneity, manifested in immune cell infiltration, stromal remodelling and epidermal dysregulation, drives divergent clinical phenotypes and variable treatment responses. The integration of spatial transcriptomics with AI‐powered analytical pipelines will definitely facilitate the identification of spatial biomarkers and molecular subtypes, allowing patient stratification based on cellular and spatial phenotypes rather than traditional histopathological classifications.

## CONCLUSIONS

**FIGURE 2:**
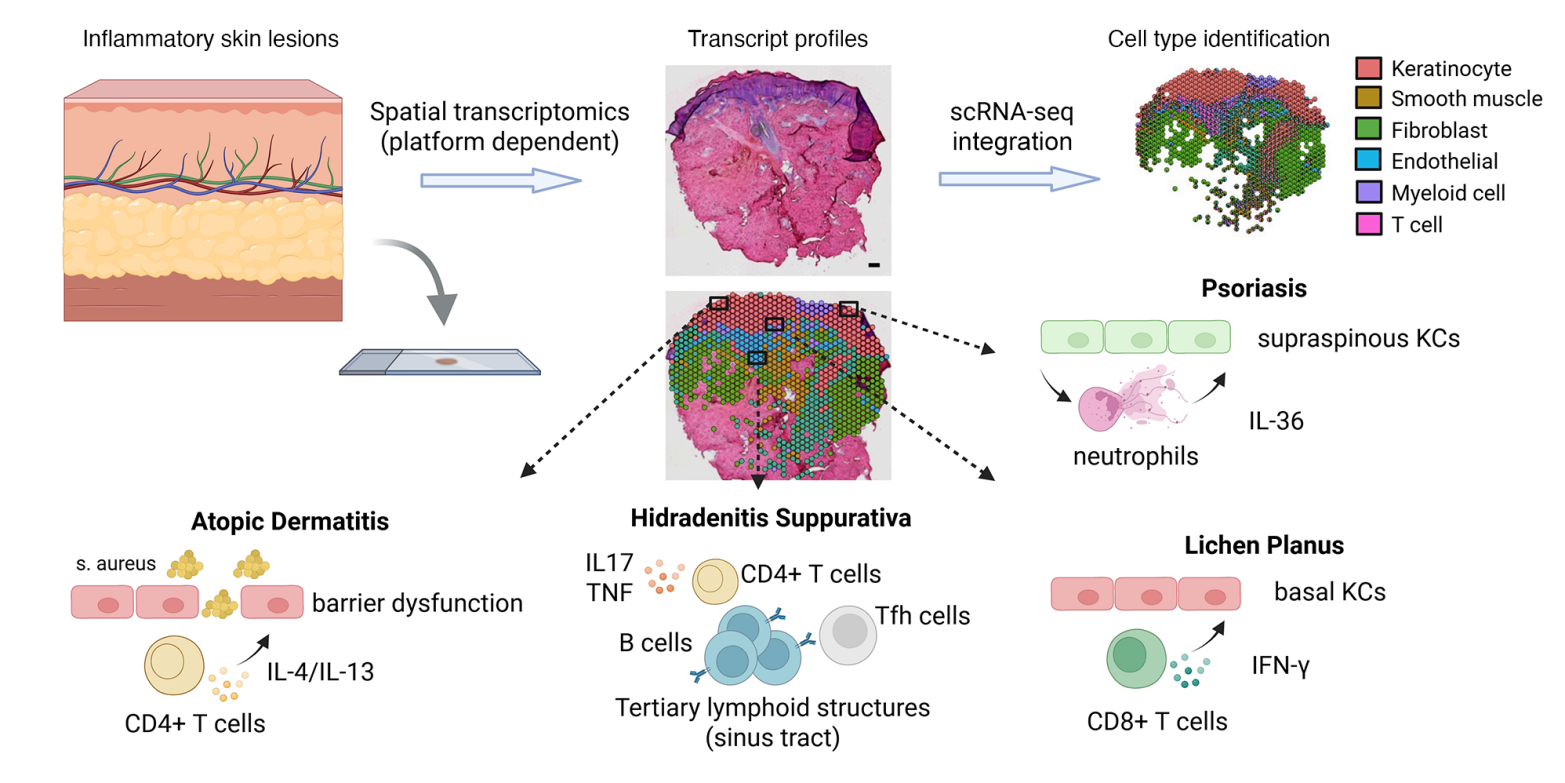
Spatial transcriptomics reveals immune–stromal interactions in inflammatory skin diseases. Integration of single‐cell and spatial transcriptomic data uncovers disease‐specific inflammatory niches, highlighting immune–stromal interactions in atopic dermatitis, hidradenitis suppurativa and lichen planus.

Spatial transcriptomics is reshaping our understanding of inflammatory skin diseases by enabling unprecedented resolution of the spatial architecture of gene expression and immune–stromal interactions (Figure 2). Despite technical limitations—such as suboptimal transcript capture efficiency and incomplete coverage of the transcriptome—ongoing advances and integration with complementary technologies like spatial proteomics, metabolomics and chromatin accessibility profiling are rapidly expanding its analytical power. Moreover, the incorporation of AI‐based tissue reconstruction and modelling pipelines holds transformative potential for biomarker discovery and patient stratification. As spatial multi‐omics continues to evolve, it promises to bridge the gap between molecular pathology and clinical dermatology, ultimately driving precision medicine forward.

## AUTHOR CONTRIBUTIONS

Conceptualization: J.E.G., R.J.; Writing – original draft preparation: R.J., Z.F.; writing – review and editing: R.J., L.C.T., J.E.G.

## FUNDING INFORMATION

This work was supported by NIH grants R01‐AI183620 (JG), R01‐AI179251 (JG), R01‐AI130025 (JG), P30‐AR075043 (JG), PO1‐AI179251 (JG) and the Taubman Medical Research Institute (JG).

## CONFLICT OF INTEREST STATEMENT

J.E.G. has served as a consultant to AbbVie, Eli Lilly, Almirall, Celgene, BMS, Janssen, Prometheus, TimberPharma, Galderma, Novartis, MiRagen, AnaptysBio and has received research support from AbbVie, SunPharma, Eli Lilly, Kyowa Kirin, Almirall, Celgene, BMS, Janssen, Prometheus and TimberPharma. L.C.T. has received support from Galderma and Janssen. The rest of the authors declare that they have no relevant conflicts of interest.

## ETHICAL APPROVAL

Not applicable.

## ETHICS STATEMENT

Not applicable.

## Data Availability

Data sharing is not applicable to this article as no new data were created or analyzed in this study.
